# A tumor-restricted glycoform of podocalyxin is a highly selective marker of immunologically cold high-grade serous ovarian carcinoma

**DOI:** 10.3389/fonc.2023.1286754

**Published:** 2023-12-21

**Authors:** Julyanne Brassard, Michael R. Hughes, Pamela Dean, Diana Canals Hernaez, Shelby Thornton, Allyson C. Banville, Julian Smazynski, Mary Warren, Kevin Zhang, Katy Milne, C. Blake Gilks, Anne-Marie Mes-Masson, David G. Huntsman, Brad H. Nelson, Calvin D. Roskelley, Kelly M. McNagny

**Affiliations:** ^1^ School of Biomedical Engineering, University of British Columbia, Vancouver, BC, Canada; ^2^ Department of Cellular and Physiological Sciences, University of British Columbia, Vancouver, BC, Canada; ^3^ Molecular and Advanced Pathology Core (MAPcore), University of British Columbia, Vancouver, BC, Canada; ^4^ British Columbia Cancer Agency, Victoria, BC, Canada; ^5^ Department of Pathology and Laboratory Medicine, University of British Columbia, Vancouver, BC, Canada; ^6^ Centre de Recherche du Centre hospitalier de l’Université de Montréal, Montreal, QC, Canada; ^7^ Department of Molecular Oncology, University of British Columbia, Vancouver, BC, Canada

**Keywords:** podocalyxin, glycoepitope, immune cold, high-grade serous ovarian carcinoma, immunotherapy

## Abstract

**Introduction:**

Targeted-immunotherapies such as antibody-drug conjugates (ADC), chimeric antigen receptor (CAR) T cells or bispecific T-cell engagers (eg, BiTE^®^) all aim to improve cancer treatment by directly targeting cancer cells while sparing healthy tissues. Success of these therapies requires tumor antigens that are abundantly expressed and, ideally, tumor specific. The CD34-related stem cell sialomucin, podocalyxin (PODXL), is a promising target as it is overexpressed on a variety of tumor types and its expression is consistently linked to poor prognosis. However, PODXL is also expressed in healthy tissues including kidney podocytes and endothelia. To circumvent this potential pitfall, we developed an antibody, named PODO447, that selectively targets a tumor-associated glycoform of PODXL. This tumor glycoepitope is expressed by 65% of high-grade serous ovarian carcinoma (HGSOC) tumors.

**Methods:**

In this study we characterize these PODO447-expressing tumors as a distinct subset of HGSOC using four different patient cohorts that include pre-chemotherapy, post-neoadjuvant chemotherapy (NACT) and relapsing tumors as well as tumors from various peritoneal locations.

**Results:**

We find that the PODO447 epitope expression is similar across tumor locations and negligibly impacted by chemotherapy. Invariably, tumors with high levels of the PODO447 epitope lack infiltrating CD8^+^ T cells and CD20^+^ B cells/plasma cells, an immune phenotype consistently associated with poor outcome.

**Discussion:**

We conclude that the PODO447 glycoepitope is an excellent biomarker of immune “cold” tumors and a candidate for the development of targeted-therapies for these hard-to-treat cancers.

## 1 Introduction

Ideal cancer therapies directly target tumor cells and spare healthy tissues resulting in increased efficacy and reduced side-effects. Antibody-drug conjugates (ADC), chimeric antigen receptor (CAR) T cells or bispecific T-cell engagers (eg, BiTE^®^) are examples of attempts to exploit these properties of tumor-targeting cancer therapies. The choice of the epitope is crucial in reaching this level of cancer specificity but, unfortunately, true tumor-specific epitopes are extremely rare, especially in solid lesions (1).

Podocalyxin (PODXL) is a CD34-related stem cell sialomucin, expressed by induced pluripotent stem cells (iPSCs) and a range of embryonic tissues during development, but much more restricted to vascular endothelia and kidney podocytes in adult (2–5). This family of proteins act as blockers of cell adhesion and enhancers of cell mobility (4) and we and others have shown that PODXL, in particular, is upregulated by a wide array of tumors and its expression consistently correlates with poor outcome (6–9). We and others previously showed that inactivation of PODXL in cancer cell lines cripples their ability to form tumors in xenografted mice and correlates with a decrease in tumor-initiating cell (TIC) frequency *in vivo* (10–12). We also showed that antibodies targeting a functional domain of PODXL can block tumor growth in xenografts (10). Nevertheless, the expression of the PODXL core protein by normal podocytes and vascular endothelia presents a potential barrier to further antibody-targeted therapies due to the possibility of normal tissue toxicity.

Intriguingly, dysregulation of glycosylation pathways are known to frequently occur during cancer development and this can lead to the generation of tumor-associated carbohydrate antigens (TACA) which represent a potential array of tumor-specific neo-epitopes (13, 14). Taking advantage of this peculiarity of neoplastic cells, we recently developed a novel monoclonal antibody (mAb), named PODO447, that targets a glycopeptide epitope of PODXL expressed on cancer cells, but is absent on normal, PODXL-expressing podocytes and vascular endothelia (15, 16). This glycoepitope comprises a peptide sequence within the mucin domain of PODXL together with a core 1 disaccharide (Galβ1-3GalNAc-α-1-O-Thr/Ser), also called the “T-antigen” (16). Since the core protein of PODXL is aberrantly expressed by many human tumors and its expression is consistently linked to poor prognosis (reviewed in (4, 8, 14, 17, 18)), this PODO447 epitope serves as an excellent candidate for the development of targeted cancer immunotherapies. Indeed, we recently documented the effectiveness of a PODO447-directed ADC against human xenografted tumors as proof-of-principle to further support this concept (16). Intriguingly, in the case of high-grade serous ovarian carcinoma (HGSOC), we previously found that roughly 65% of these tumors express the PODO447 epitope (15). Effective therapies for HGSOC are relatively limited and these tumors are typically controlled through debulking surgery combined with perioperative platinum-based chemotherapy. Unfortunately, they frequently recur as incurable disease within the first five-years (19). Moreover, tumor lesions frequently appear in a variety of anatomical locations that include the site of origin in the distal portion of the fallopian tubes and the ovary as well as other abdominal sites including the peritoneum, omentum and the small and large intestines (19, 20).

HGSOC are generally considered to be “immune-cold” as the lesions often display few tumor-infiltrating lymphocytes (TILs). Indeed, this likely explains their generally poor response rate to immune checkpoint inhibitors (21, 22). Nevertheless, a fraction of HGSOC patients present tumors with infiltrating CD8^+^ T cells in combination with intraepithelial or stromal CD20^+^ cells, a marker of B cells and plasma cells. Importantly, patients with dual infiltration exhibit statistically better survival/outcome than patients presenting with immune-cold tumors or tumors that are only infiltrated by CD8^+^ T cells (23, 24).

Here we evaluate the expression of the PODO447 epitope and immune infiltrates using four different HGSOC patient cohorts that include pre-chemotherapy, post-neoadjuvant chemotherapy (NACT) and relapsing tumors as well as tumors from various peritoneal locations. We find that the PODO447 epitope is expressed across tumor locations and expression is retained after standard chemotherapy. Furthermore, we find that, consistently, tumors with high levels of the PODO447 epitope exhibit poor infiltration with CD8^+^ and CD20^+^ cells. Our data suggest that targeting the PODO447 epitope may offer a novel opportunity to treat immune-cold HGSOC tumors as well as those for whom standard-of-care therapies have failed.

## 2 Materials and methods

### 2.1 High-grade serous ovarian carcinoma patient cohorts

This study was performed under the University of British Columbia Research Ethics Review Board protocol H21-01513 and includes data from four different cohorts described in **Table 1**. Cohort A (GPEC 08-001) was obtained from the Genetic Pathology Evaluation Centre TMA Database (Vancouver, Canada) and includes cases from 2000 to 2007 taken at surgery. Cohort B (CHUM THT3) was obtained from the Terry Fox research institute (TFRI) Canadian Ovarian Experimental Unified Resource (COEUR) and includes cases from 1998 to 2011 taken at surgery at different time points from the same patient. Cohort C (CHUM pre/post-chemotherapy) tumor samples were obtained from patients who underwent surgery in the CHUM Department of Gynecologic Oncology between 1993 and 2014. Cohort D (IROCPROS5) consists of untreated surgical samples collected through BC Cancer’s Tumor Tissue Repository from 2007 to 2014.

**Table 1:** Main characteristics of the study population.

		Cohorts			
		GPEC 08-001	CHUM THT3	CHUM (pre/post chemo)	IROCPROS5
Identification		A	B	C	D
TMA data	# cores per tumor	**2**	**3**	**2-3**	**4** 2 tumor, 2 stroma area
	core size	0.8 mm	0.8 mm	0.8 mm	0.6 mm
	# tumors per patient	1	3 - 10	1	1 - 2
Scorable tumors for PODO447 staining		Patients/tumors n = 176 • Pre-chemo: 145 • Post-chemo: 31	Patients n = 14 Tumors n = 99 • Pre-chemo: 62 • Post-chemo: 37	Patients/tumors n = 124 • Pre-chemo: 71 • Post-chemo: 53	Patients n = 52 Tumors n = 61 • Ovary: 33 • Omentum: 26 • Fallopian Tube:1 • Pelvic Mass: 1
Age- year ± SD		Age at surgery 61.98 ± 11.96	Age at diagnosis 58.64 ± 10.27	Age at diagnosis 60.45 ± 9.77	Age at surgery 64.23 ± 11.25
FIGO stage		Unknown: 16 I: 11 II: 13 III: 117 IV: 19	III: 14	Unknown: 2 I: 2 II: 6 III: 99 IV: 15	I: 1 II: 5 III: 40 IV: 6
Chemotherapeutic agent		No chemo (145) Taxol/carboplatin (21) Carboplatin/paclitaxel (4) Carboplatin/taxotere (1) Cisplatin/topotecan (1) Carboplatin/paclitaxel/epirubicin (1) Unspecified platinum/taxane (1) Unknown (2)	No chemo (1) Taxol/carboplatin (8) Taxol/cisplatin (2) Carboplatin/taxotere (1) Cisplatin/topotecan (1) Carboplatin/paclitaxel (1)	No chemo (71) Taxol/carboplatin (36) Paclitaxel (1) Doxorubicin (1) Cisplatin/topotecan (1) Unknown (13)	No chemo (52)
Time since chemo started (months ± SD)			Min 3; Max 81 Mean: 30.54 ± 28.15		N/A
Time since chemo ended (months ± SD)			Min 0; Max 77 Mean: 26.77 ± 27.92		N/A
Number of chemo cycles ± SD		Min 3; Max 9 Mean: 3.77 ± 1.3	Min 4; Max 8 Mean: 5.69 ± 1.03		N/A

SD: standard deviation; N/A: non-applicable.

*Includes only patients with data available for PODO447 IHC staining.

### 2.2 Histology

PODO83, a mAb that recognizes the PODXL protein in both normal and tumor tissues independently of its glycosylation status was used to monitor expression of the PODXL core protein (10, 15). PODO83, tumor-specific PODO447 and Palivizumab (negative control) DAB (3,3’-Diaminobenzidine) immunohistochemistry (IHC) staining protocols were performed on consecutive sections of Cohort A, B and C tumor microarray (TMA) slides. For Cohort D, only PODO83 and PODO447 staining protocols were performed. The threshold over which a tumor was considered positive for PODO83 or PODO447 was set as five percent of positive tumor cells. Cohort D immune infiltration analysis was performed using a multiplex three-color IHC panel to detect CD3, CD8 and CD20. Cohort A multiplex panel to detect CD8, CD20 and Pan-cytokeratin (Pan-CK) markers and Cohort D multiplex panel to detect CA125, Mesothelin (MSLN), Folate receptor alpha (FOLRA) and Pan-CK markers were performed using the fluorescent Opal multiplex technology. Additional information regarding the detailed staining protocols and the concentration of antibodies used can be found in **Supplementary Methods** and **Supplementary Table S1 & S2**.

### 2.3 Histology analysis

Stained TMA slides were scanned and analyzed using QuPath software (v0.4.3) (25) to determine the percentage of positive tumor cells (PODO83, PODO447, CA125, MSLN and FOLRA), the tumor H-score (PODO83, PODO447) or the number of immune cells in the tumor or stroma areas (CD8, CD3, CD20).

#### 2.3.1 Detailed QuPath analysis workflow

##### PODO83 and PODO447 DAB staining analysis (Cohort A, B, C & D)

Because the TMAs for the different cohorts were stained at different time points and in different research facilities, an individual QuPath analysis using cohort-specific parameters was done for each cohort (same analysis for PODO83 and PODO447). A training image that includes representative cores from the TMA was created and then used to determine the optimal parameter for the detection of the background, hematoxylin, and DAB staining. The training image was then used to determine the optimal parameters for the automated cell detection command. An object classifier was then trained to distinguish between stromal and tumor areas based on manual annotation of the training image. An automated quantification of DAB staining was then applied to the tumor area using three different DAB intensity thresholds (1^st^ threshold = DAB-positive, low intensity, 2^nd^ = moderate intensity, 3^rd^ = high intensity). A script of the analysis performed on the training image was generated and applied to all the TMA cores. QuPath measurements that include number of negative cells, number of positive cells for each threshold and tumor H-score were used for subsequent analysis.

##### 2.3.1.2 CD3, CD8 and CD20 brightfield multiplex analysis (Cohort D)

QuPath analyses were similar to the ones described in the previous section, but instead of performing an automated annotation of the immune populations, following the application of the tumor vs stroma classifier to all the TMA cores, immune cell populations were manually counted. CD3^+^CD8^+^, CD3^+^CD8^-^, CD3^-^CD20^+^ were identified as CD8^+^ T cells, CD4^+^ T cells and CD20^+^ B cells respectively. Immune cells surrounded by three or more cancer cells were identified as intra-tumoral, while the remaining were labeled as stromal immune cells.

##### 2.3.1.3 Opal fluorescent multiplex analysis (Cohort A & D)

Using an approach described previously, cell detection and classifier scripts were used to identify individual cells and classify areas as “tumor epithelium” (pan-CK+) or “stroma” (pan-CK-). For CA125, MSLN and FOLRA analyses, a single intensity threshold was determined to classify tumor cells (pan-CK+) as positive or negative for the marker of interest. CD8^+^ and CD20^+^ cells were identified as CD8 T cells and CD20 B cells/plasma cells respectively and were automatically labeled as intra-tumoral or stromal based on the area classifier (surrounded by pan-CK+ vs - cells).

#### 2.3.2 Data normalization and tumor quantification

All the QuPath analyses (25) were performed twice leading to two data sets for each core. Percentage of positive tumor cells (PODO83, PODO447, CA125, MSLN, FOLRA) was obtained by calculating the summation of all positive tumor cells from each core in both sets of data and dividing this result by the total number of tumor cells.

To calculate a single tumor H-score value for PODO83 and PODO447 staining, the tumor H-score values for each core, from the same tumor, in each data set were normalized by multiplying the tumor H-score value of a specific core by the total number of cancer cells in this core. Then, the summation of these normalized tumor H-score values was divided by the total number of cancer cells in all the cores from both data sets.

Number of immune cells was calculated by dividing the summation of the number of the specific immune cell types in each core of both data sets by the total stroma or tumor area (cells/μm^2^) for all the cores from the same tumor in both data sets. This number was then multiplied by the average stroma or tumor area for one core (μm^2^), leading to a normalized number of immune cells per core.

### 2.4 Statistical analysis

Statistical analyses were performed using GraphPad Prism (v10.0.0). Statistical tests performed for each figure panel are mentioned in their associated figure legend.

## 3 Results

### 3.1 Heterogeneous expression of the PODO447 tumor glycoepitope in HGSOC

**Figure 1:**
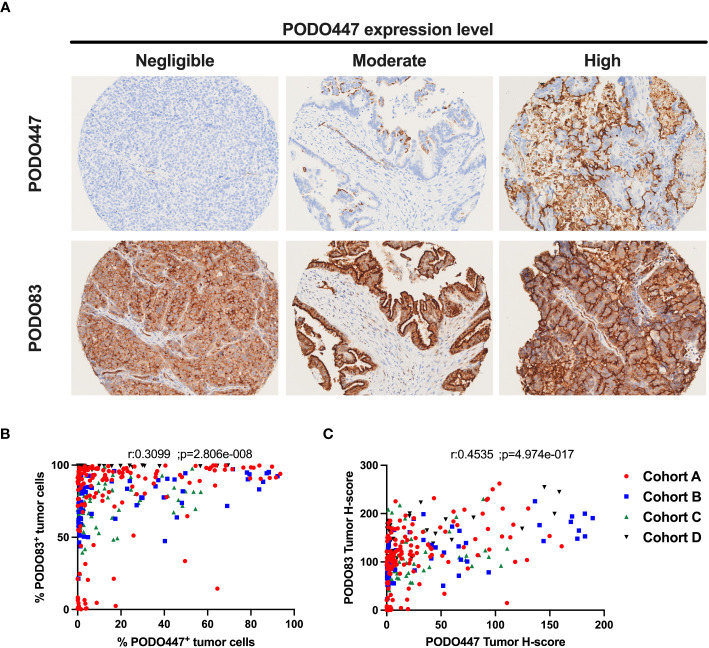
Heterogeneous expression of the PODO447 tumor glycoepitope across high-grade serous ovarian carcinoma. **(A)** Representative PODO447 and PODO83 immunohistochemistry (IHC) staining in HGSOC tumors. PODO447 staining was classified as negligible, moderate and high (top row) with matching cores for PODO83 staining (bottom row) **(B, C)** Spearman’s correlation of PODO447 and PODO83 expressing tumors presented as **(B)** the percentage of positive tumor cells or **(C)** the tumor H-score. All specimens analyzed were from naïve ovarian tumors (no-chemotherapy) in cohort A (red), B (blue), C (green) and D (black).

As a first step to determining the proportion of patients that could benefit of a PODO447-based immunotherapy, we evaluated the expression of the PODXL core protein (PODO83 immunoreactivity) and the PODO447-reactive epitope across multiple HGSOC tumors by IHC using tumor samples from the four different HGSOC patient arrays described in **Table 1** (different colors **Figures 1B, C**). Consistent with previous publications (15), we found that virtually all tumors analysed (97.4% combined data of the four cohorts) were uniformly positive for the PODXL core protein (PODO83^+^) (**Table 2**). We found that most of these tumors (57.6% combined data of the four cohorts) were also positive for the expression of the PODO447 glycoepitope (**Table 2**) though with a more restricted expression pattern in a subset of cells within each tumor. While the measure of the positivity using a single threshold is useful in estimating the proportion of patients that express PODXL epitopes, this method fails to fully capture the variability in the proportion of tumor cells that express PODXL epitopes or their level of expression (intensity of the staining, **Figure 1A**). In general, there was a positive correlation between PODO83 and PODO447 expression (**Figure 1B**). However, because PODO83 was expressed in most tumor cells (the majority approaching 100% of the cells) the full extent of this positive correlation was only exposed when the expression of both epitopes was measured using the tumor H-score (**Figure 1C**).

**Table 2:** PODO83 and PODO447 immunoreactivity in HGSOC patient cohorts.

HGSOC cohorts		PODO83				
		Scorable cases	% positive cases		Tumor H-score	
			Positive (% ± 95%CI^a^)	Delta^a^	Median ± 95%CI	Delta^b^
GPEC 08-001 (A)	Pre-chemo Post-chemo Total	14 29 173	132 (**91.7%** ± 86.0; 95.2) 29 (**100%** ± 88.0; 100) 161 (**93.1%** ± 88.3; 96.0)	8.3% p= 0.2259	113.3 ± 105.7; 121.9) 157.6 ± 129.9; 193.3 121.2 ± 111.6; 130.5	44.3 **p<0.001**
CHUM THT3* (B)	Pre-chemo Post-chemo Total	62 35 97	62 (**100%** ± 94.2; 100) 35 (**100%** ± 90.1; 100) 97 (**100%** ± 96.2; 100)	0%	106.6 ± 93.73; 132.2 93.06 ± 67.73; 118.6 101.05 ± 88.95; 119.3	-13.55 **p=0.0491**
CHUM (pre/post chemo) (C)	Pre-chem Post-chem Total	74 66 140	74 (**100%** ± 95.1; 100) 66 (**100%** ± 94.5; 100) 140 (**100%** ± 97.3; 100)	0%	99.44 ±89.60; 110.0 94.27 ± 85.59; 106.9 96.71 ± 89.60; 104.4	-5.179 p=0.5728
IROCPROS5 (D)*	Pre-chemo (Total)	58	58 (**100%** ± 93.8; 100)	⌀	172.3 ± 165.0; 177.3	⌀
All four cohorts	Pre-chemo Post-chemo Total	338 130 468	326 (**96.5%** ± 93.9; 98.0) 130 (**100%** ± 97.1; 100) 456 (**97.4%** ± 95.6; 98.5)	3.5% p= 0.0643	118.6 ± 111.1; 126.9 105.0 ± 94.7; 117.3 113.6 ± 108.0; 121.4	-13.62 p=0.0550
HGSOC cohorts		PODO447				
		Scorable cases	% positive cases		Tumor H-score	
			Positive (% ± 95%CI^a^)	Delta^a^	Median ± 95%CI	Delta^b^
GPEC 08-001 (A)	Pre-chemo Post-chemo Total	145 31 176	86 (**59.3%** ± 51.2; 67.0) 22 (**71.0%** ± 53.4; 83.9) 108 (**61.3%** ± 54.0; 68.2)	11.7% p= 0.3141	10.14 ± 5.92; 15.91 12.36 ± 7.69; 19.7 11.38 ± 7.69; 15.28	2.22 p=0.5184
CHUM THT3* (B)	Pre-chemo Post-chemo Total	62 37 99	32 (**51.6%** ± 39.4; 63.6) 22 (**59.5%** ± 43.5; 73.7) 54 (**54.5%** ± 44.7; 64.0)	7.9% p= 0.550	7.40 ± 2.52; 41.34 8.14 ± 3.89; 23.38 8.02 ± 3.96; 24.45	0.7445 p=0.984
CHUM (pre/post chemo) (C)	Pre-chemo Post-chemo Total	71 53 124	39 **(54.9%** ± 43.3; 66.0) 34 (**64.2%** ± 50.7; 75.7) 73 (**58.9%** ± 50.1; 67.1)	9.3% p= 0.848	8.13 ± 3.36; 16.99 15.64 ± 5.43; 21.28 9.97 ± 5.45; 16.99	7.505 p=0.1933
IROCPROS5 (D)*	Pre-chemo (Total)	61	30 (**49.2%** ± 37.1; 61.4)	⌀	8.33 ± 3.49; 21.22	⌀
All four cohorts	Pre-chemo Post-chemo Total	339 121 460	187 (**55.2%** ± 49.8; 60.4) 78 (**64.5%** ± 55.61; 72.43) 265 (**57.6%** ± 53.0; 62.0)	9.3% ± p= 0.0949	8.50 ± 6.39; 13.63 11.97 ± 8.02; 17.92 9.53 ± 7.69; 13.21	3.47 p=0.2600

* Includes tumors from different peritoneal locations.

CI^a^: Confidence interval calculated using Wilson/Brown test;

Delta^a^: %Post-chemo – %Pre-chemo (p-value calculated using Chi-square with Yates’ correction);

Delta^b^: Median Post-chemo – Median Pre-chemo (p-value calculated using Mann Whitney test).

### 3.2 PODO447 immunoreactivity is similar in ovarian or distal tumors

**Figure 2:**
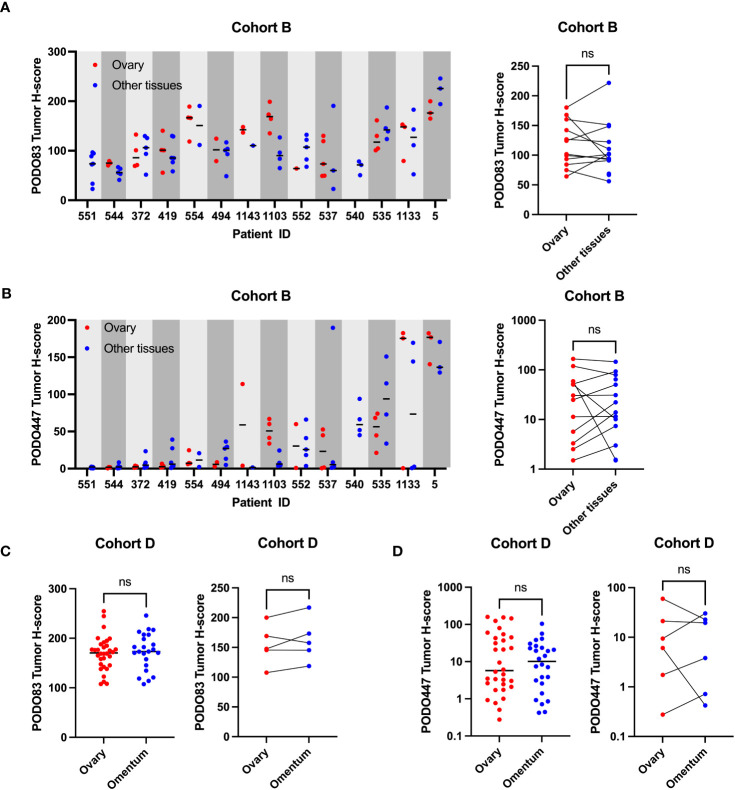
PODO447 tumor glycoepitope is also expressed on non-ovarian tumoral lesions in HGSOC patients. **(A)** PODO83 and **(B)** PODO447 tumor H-score in ovarian tumors (red) compared to tumors in other tissues (blue) presented as individual values and median for each patient (left panel) or mean of all the ovarian vs mean of all the tumors from other tissues for the same patient (right panel). **(C)** PODO83 and **(D)** PODO447 tumor H-score compared between ovarian tumors (red) and omentum tumors (blue) in unpaired (left panel) or paired samples (right panel). Data for AB) are from Cohort B (including naïve and post-chemotherapy samples) and data for CD) are from Cohort D. ns= non-significant; Wilcoxon (paired-data) and Mann-Witney (unpaired-data) tests.

In addition to ovarian lesions that originally arose in the fallopian tubes, HGSOC patients frequently have tumors that have spread to distal locations within the abdomen including the omentum, peritoneum, pelvis and intestines (19, 20). We therefore evaluated the expression of the PODXL core protein and the PODO447 glycoepitope collected from different anatomical sites from the same patients (Cohort B). We found no clear trend toward increased or decreased expression of PODO83 or PODO447 when comparing a range of anatomical sites (**Figures 2A, B**). In fact, analyses of the mean tumor H-score from all the tumors in the same group (ovary or other tissues) revealed no significant differences between groups (**Figures 2A, B**; right panels). Individual patient analyses instead show that PODO83 and PODO447 expressions are similar across tumor locations from the same patient resulting in a significant patient pairing (Spearman; PODO83: p = 0.0200, PODO447: p = 0.0375) (**Figures 2A, B**). To confirm these results, PODO83 and PODO447 expression was also compared between tumors collected from the ovary and the omentum from Cohort D and again no significant differences were observed in unpaired data and patient-paired data (**Figures 2C, D**). Importantly, these results suggest that, if developed as a therapy, PODO447 would be capable of targeting HGSOC lesions in all anatomical locations.

### 3.3 The PODO447 epitope persists following chemotherapy

**Figure 3:**
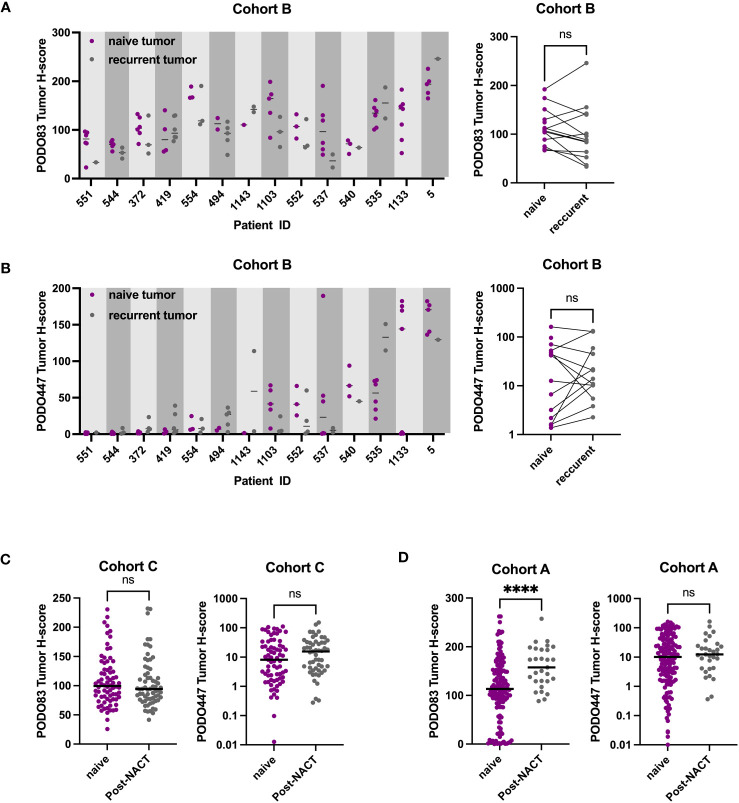
PODO447 expression persists following chemotherapy treatment. **(A)** PODO83 and **(B)** PODO447 tumor H-score in naïve tumors (pre-chemotherapy: purple) compared to recurrent post-chemotherapy tumors (gray) presented as individual values with the median for each patient (left panel) or mean of all the naïve vs mean of all the recurrent tumors for the same patient (right panel). **(C, D)** PODO83 and PODO447 tumor H-score compared between naïve (purple) and post-chemotherapy (gray) in unpaired tumor samples from **(C)** cohort C and **(D)** cohort A. ns = non-significant; ****p <0.0001; Wilcoxon (paired-data) and Mann-Witney (unpaired-data) tests.

Previous reports suggest that the expression level of some tumor epitopes can vary dramatically through the course of anti-cancer treatment (26–28). To determine whether this is also the case with PODXL epitopes, PODO83 and PODO447 tumor H-scores were evaluated in pre-chemotherapy (naïve), post-neoadjuvant chemotherapy (NACT) and recurrent post-chemotherapy tumors in three different cohorts (**Figure 3** and **Table 2**). Individual patient analyses (Cohort B) revealed some modulation in both PODO83 and PODO447 expression between naïve and post-chemotherapy recurrent tumors, but these modulations include both up- and down-regulation resulting in no overall significant trends (**Figures 3A, B**). Similar results were observed in un-paired tumor data from Cohort C, with no significant difference in PODO83 or PODO447 expression between naïve and post-NACT tumors (**Figure 3C**). In Cohort A, the expression of PODO83 is significantly increased in post-NACT samples compared to unpaired naïve tumors (**Figure 3D**), but this upregulation was not reflected in the intensity of the expression of the PODO447 glycoepitope and was stable between groups (**Figure 3D**). Interestingly, despite this absence of PODO447 tumor H-score modulation, in all three cohorts (A, B, C) the proportion of tumors that are PODO447 positive and the median PODO447 tumor H-score are slightly higher in patients that previously received chemotherapy, although this trend did not reach statistical significance (**Table 2**). Importantly, the persistence of PODO447 expression in post-NACT and post-chemotherapy recurrent tumors suggest that PODO447-based therapies could also be of value as a second-line treatment.

### 3.4 HGSOC tumors expressing a high level of the PODO447 epitope exhibit an immune-cold phenotype

**Figure 4:**
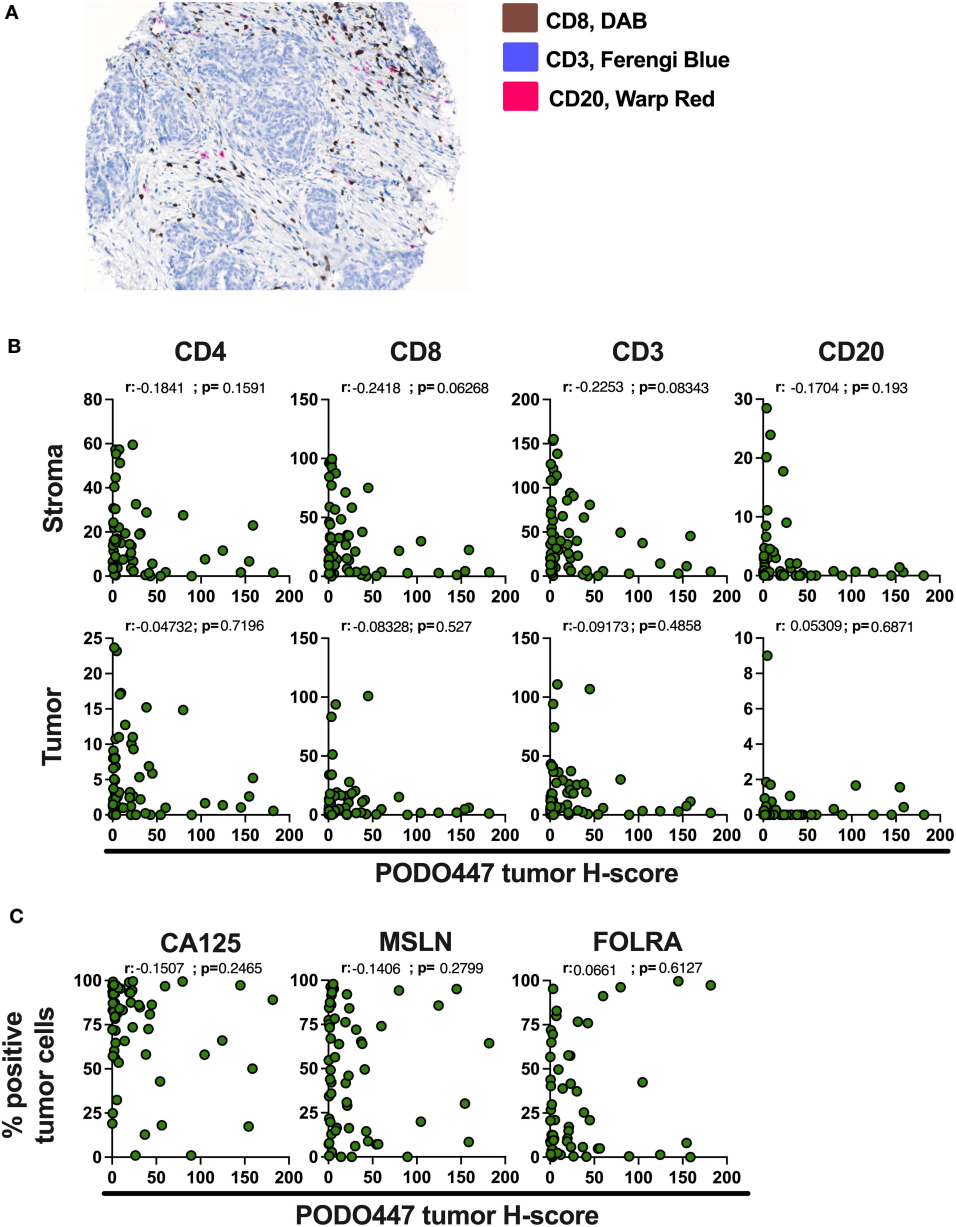
High expression of the PODO447 epitope is associated with a lower level of immune infiltrates in HGSOC. **(A)** Representative image of the multiplex IHC staining for CD8 (DAB), CD3 (Ferengi Blue) and CD20 (Warp Red). **(B)** Spearman’s correlation between PODO447 tumor H-score and the number of CD4^+^, CD8^+^ and CD3^+^ T cells and CD20^+^ cells in the stroma area (upper panel) or the tumor area (bottom panel). **(C)** Spearman’s correlation between PODO447 tumor H-score and the percentage of CA125, Mesothelin (MSLN) and folate receptor alpha (FOLRA) positive tumor cells. Data are from Cohort D.

Immune infiltration of solid tumors is an important predictor of disease outcome and response to some cancer immunotherapies (29). In the case of HGSOC, infiltration by a combination of CD8^+^ T cells and CD20^+^ B cells/plasma cells has been associated with improved survival (23, 24). Accordingly, we evaluated the expression of the PODO447 tumor glycoepitope and the number of CD4^+^, CD8^+^ and CD3^+^ T cells and CD20^+^ B cells/plasma cells *via* multiplex IHC staining of Cohort D (**Figure 4A**). In tumors where the stroma-rich areas were highly infiltrated with these lymphocytes, we found proportionally lower levels of PODO447 epitope expression in tumor cells (**Figure 4B**, upper panel). Conversely, high PODO447-expressing tumors were consistently poorly infiltrated by all three immune populations (**Figure 4B**, upper panel). The same trends were observed when the tumor-rich stroma-free regions for CD4^+^ and CD8^+^ T cells were examined (**Figure 4B**, bottom panel). In contrast, overall, there was a low number of CD20^+^ cells within the tumor-regions of all the specimens in Cohort D regardless of their PODO447 expression status (**Figure 4B**, bottom panel, right graph). To better visualize these differences in immune infiltration, we segregated tumors into three groups based on their PODO447 tumor H-score (Low <5, Med 5 to 50, High >50). In both the stroma and the tumor areas, a clear trend emerged toward lower infiltration for all the immune cell populations in the PODO447 high group (except for CD20^+^ cells in tumor-rich areas). This is exemplified by the lower median in the PODO447 high group compared to the two other groups (**Supplementary Figure 1A**). This association between PODO447 glycoepitope expression and low immune infiltrates is not directly caused by an increase in overall PODXL core protein expression in the PODO447 high tumors, as we find no clear associations between PODXL expression (i.e. PODO83 immunoreactivity) and immune infiltration in the stroma or tumor areas (**Supplementary Figure 1B**). Instead, it argues for a more selective association with the tumor-specific glycosylation of PODXL that is recognized by the PODO447 antibody. To provide additional information about their immune profile, tumors were segregated into three immune-phenotypes groups based on both CD8^+^ cell and CD20^+^ cell infiltration as well as the localization of these immune cells. The criteria used to describe these immune-phenotypes groups (immune-desert, -excluded and -inflamed) are presented in **Supplementary Figure 2A**. Although, no significant differences were observed, the highest PODO447 tumor H-score median was in the immune-desert group (**Supplementary Figure 2B**). Again, this was not due to a general overexpression of the core PODXL protein as PODO83 level was not elevated in the immune-desert group (**Supplementary Figure 2B**). Interestingly, while most PODO447^Low/Med^ expressing tumors were either immune-excluded or inflamed, the majority of the PODO447^High^ expressing tumors displayed an immune-desert phenotype (**Supplementary Figure 2C**).

We next attempted to correlate expression levels of PODO447 immunoreactivity with other well-characterized ovarian tumor markers including CA125 (a mucin 16 epitope), mesothelin (MSLN) and folate receptor alpha (FOLRA), three tumor antigens expressed by HGSOC that are under development as therapeutic targets for the treatment of ovarian cancer (30–36). None of these markers showed a clear correlation with PODO447 expression (**Figure 4C**). Furthermore, and in contrast with PODO447 expression, none of these tumor antigens was associated with an overall lower level of immune infiltration (**Suppl Figures 3A-C**). In aggregate, our data indicate that the PODO447 epitope is a highly selective biomarker for distinguishing immunologically cold tumors and could serve as an alternative target for the treatment of these immune-cold HGSOC tumors and provide a higher degree of specificity than CA125, MSLN and FOLRA.

### 3.5 Validation of the association between PODO447 expression and immune-cold phenotype

**Figure 5:**
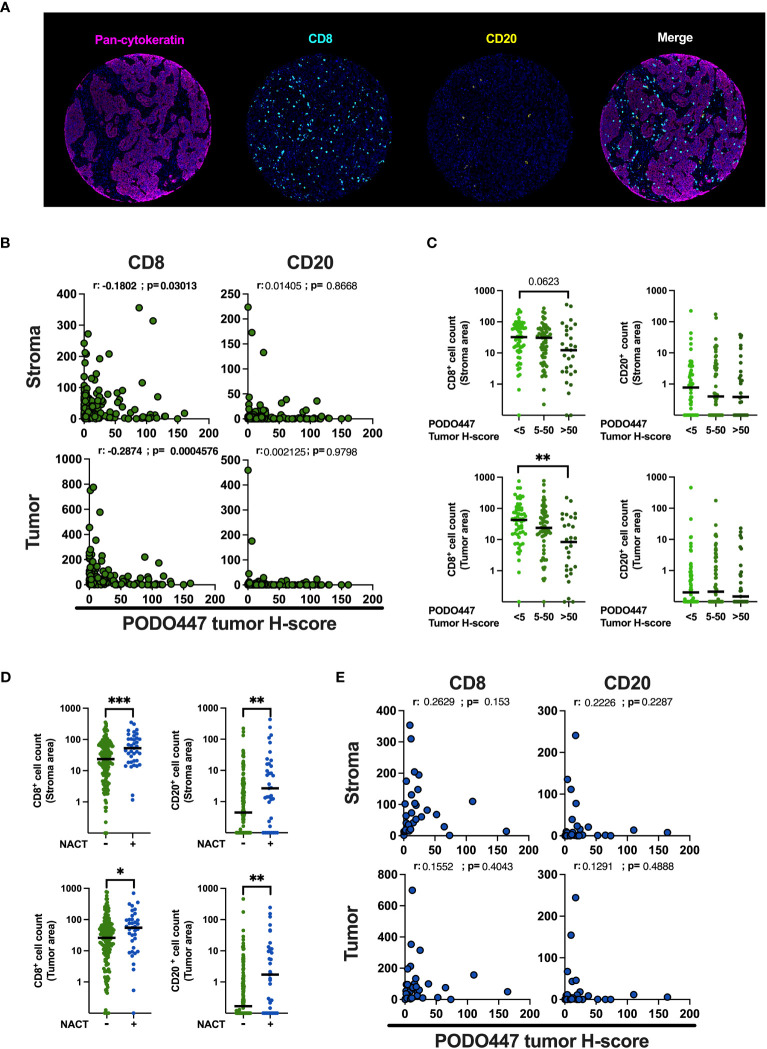
Validation of the negative association between PODO447 expression and the level of CD8^+^ cell infiltration. **(A)** Representative images of the opal multiplex immunofluorescent staining used to identify CD8^+^ (blue) cells and CD20^+^ (yellow) cells in the tumor (pan-CK+) and stroma (pan-CK-) areas. **(B, E)** Spearman’s correlation between PODO447 tumor H-score and the number of CD8^+^ cells and CD20^+^ cells in the stroma (upper panel) or the tumor (bottom panel) areas **(B)** in naïve tumors and **(E)** in tumors that previously received NACT. **(C)** Scatter plot with median comparing the number of CD8^+^ cells and CD20^+^ cells in the stroma (upper panel) or in the tumor (bottom panel) areas in naïve tumors that express low (tumor H-score <5), medium (tumor H-score 5 to 50) or high level (tumor H-score >50) of the PODO447 epitope. **(D)** Scatter plot with median comparing the number of CD8^+^ cells and CD20^+^ cells in the stroma or in the tumor areas between tumors that previously received NACT and tumors that did not. Data are from Cohort A. * p<0.05; **p<0.01; ***p<0.001 using C: Kruskal-Wallis test and D: Mann-Whitney test. Only p-values below 0.1 are displayed.

To validate the association between PODO447 immunoreactivity and low level of immune infiltration obtained in Cohort D, we optimized a multiplex opal immunofluorescent panel that includes pan-CK, to distinguish between tumor and stromal areas and CD8 and CD20 immune markers and used this panel to stain the samples from the Cohort A (**Figure 5A**). Comparison of immune infiltration with PODO447 tumor H-score were performed using PODO447 staining data from the DAB IHC staining used in previous figures. In naïve (pre-chemotherapy) tumors only, expression of the PODO447 epitope was negatively correlated with CD8^+^ cell number in both the stroma and the tumor areas (**Figures 5B, C**). Unsurprisingly, no significant correlations between PODO447 glycoepitope expression and B cell infiltration were observed in the stroma or tumor areas, likely due to the very low level of B cell infiltration overall (median<1 in all groups) (**Figures 5B, C**). In accordance with previously published studies (37–39), the level of CD8^+^ cell and CD20^+^ B/plasma cell infiltration in both stromal and tumor areas was significantly increased in tumors previously treated with NACT (**Figure 5D**). Importantly, those NACT-treated tumors that were most highly infiltrated with CD8^+^ or CD20^+^ cells in both the tumor-rich and stromal areas tended to have low levels of PODO447 epitope expression. Conversely, treated tumors that expressed high levels of the PODO447 epitope tended to have low levels of immune infiltration (**Figure 5E**). The analysis of immune-phenotype, using a similar strategy to the one presented above (**Supplementary Figure 4A**), revealed that while most tumors expressing low to intermediate level of the PODO447 epitope display an immune-inflamed phenotype, tumors expressing a high level of the PODO447 epitope fell within the immune-desert group (**Supplementary Figure 4C**). These results confirm the association between PODO447 epitope expression and an immune-cold phenotype and suggest that targeting the PODO447 epitope could be a strategy to target these hard-to-treat, immune-cold tumors both before and after chemotherapy.

## 4 Discussion

Two main objectives are being currently pursued in the development of new cancer therapies: 1) targeting tumors that are refractory to current treatments and 2) developing safer treatments with reduced side effects/toxicities compared to conventional anti-cancer therapies. Together these would permit the targeting of aggressive tumors and extend disease-free survival without diminishing quality of life. To achieve this level of tumor specificity, immune-based targeted-therapies require epitopes that are highly expressed on the surface of cancer cells and weakly expressed or absent on healthy tissues (40, 41). We previously demonstrated that the PODO447 glycopeptide epitope fulfills these criteria (15, 16).

In this study, we evaluated PODO447’s potential utility in treating HGSOC as an unmet medical need. Our results demonstrate that PODO447 epitope expression is minimally impacted by previous chemotherapy treatment and persists on recurrent tumors. Furthermore, we find that PODO447 epitope is similarly retained on tumors that spread to secondary peritoneal locations, boding well for its widespread utility for multiple disseminated tumor sites. These results also suggest that a patient with a PODO447-positive tumor at the time of the initial surgery or biopsy could still be treated with PODO447-based therapies after first-line treatment with chemotherapeutic agents and that this treatment could also be effective against distal tumor lesions.

**Figure 6:**
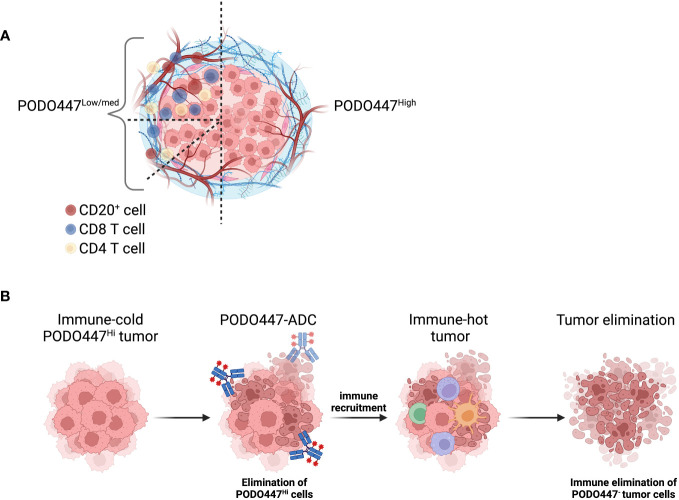
Schematic representation of PODO447 expressing tumors. **(A)** Graphical representation of the low level of immune infiltration in HGSOC that expresses a high level of the PODO447 glycoepitope and representation of all the immune-phenotypes (immune-desert, immune-excluded and immune-inflamed) observed in HGSOC tumors that express low to intermediate level of the PODO447 epitope. **(B)** Illustration of how a PODO447-ADC could be used to turn cold PODO447-expressing tumors into hot tumors and promote immune-mediated tumor elimination. Picture created using BioRender.com.

HGSOC is generally considered to be an immune-cold cancer type (21, 22). Nevertheless, better outcomes are observed in patients with tumors that are highly infiltrated with CD8^+^ T cells and CD20^+^ B/plasma cells (23, 24). Strikingly, we find that tumors expressing a high level of the PODO447 epitope are consistently poorly infiltrated with both CD8^+^ T cells and CD20^+^ cells (illustrated in **Figure 6A**). Furthermore, we found that even in NACT-treated tumors that display a general increase level of CD8^+^ T cell and CD20^+^ cell infiltration, high expression of the PODO447 epitope remains associated with poor immune infiltration (37–39). Thus, PODO447-based therapies could provide an alternative or complementary treatment option for patients that present with ‘immune-desert’ HGSOC tumors. This patient group is less likely to respond to immune checkpoint inhibitors (ICI) as the efficiency of this type of treatment is strongly associated with pre-existing immune infiltration (29). Interestingly, some targeted therapies, including ADC, can induce immune activation and turn “cold” tumors “hot” (42–44). In this regard, it is noteworthy that we have previously shown that although PODO447-ADC show efficacy against xenografted tumors in both NOD-SCID Il2Rγ^−/−^ (NSG) and Nude mice, Nude mice with residual B and NK cell activity respond much more robustly to PODO447-ADC treatment (16). These observations suggest that PODO447-ADC targeting even a subset of tumor cells may offer a strategy for turning immune-cold PODO447-expressing tumors into immune-hot tumors, and enhance their clearance or, alternatively, make them more likely to respond to ICI therapies (illustrated in **Figure 6B**).

When expression of PODO447 was compared to expression of CA125, MSLN and FOLRA (three of the most common tumor antigens currently used or under development for targeted therapies in HGSOC), only the PODO447 epitope showed a tight, inverse correlation with immune infiltration of T cells and B lineage cells. Furthermore, despite being highly expressed on HGSOC tumors, none of these epitopes are truly tumor-specific (45–47) and thus are at increased relative risk for toxic side effects through the targeting of normal tissues. Due to its exquisite tumor specificity this is less of a concern for the PODO447 epitope. Finally, we previously showed in xenograft experiments that the few remaining tumor cells following PODO447-ADC treatment are negative not only for the PODO447 glycoepitope, but also for the PODXL core protein. A similar result was obtained when PODO447 was used as a radioisotope-conjugate (manuscript in preparation). These data suggest that tumors cannot simply escape by altering PODXL glycosylation and instead suggest that the expression of this tumor glycoepitope is more intimately linked to PODXL function in these tumors (16). Because we and others have shown that loss of PODXL core protein expression cripples tumor growth *in vivo*, the loss of core protein expression likely explains the slow growth rate observed in relapsing tumors post treatment with PODO447-ADC (10–12).

The tight, inverse correlation between expression of the PODO447 epitope by a subset of tumor cells and the observed immune-cold phenotype argues strongly for a functional linkage but the mechanistic nature of this linkage remains obscure. A critical component of the tumor-specific PODO447 epitope is a disaccharide glycan structure called a “core 1 glycan” or T-antigen (16), a cryptic precursor of extended O-mucin glycosylation present in normal tissues (48). Importantly, PODO447 only recognizes this glycan in the context of a PODXL core protein poly-peptide as this epitope is not on any other tumor-associated sialomucins (16). Lectins are a large family of glycan-binding proteins and several classes of lectins such as C-type lectins, siglecs or galectins are known to influence anticancer immunity by detecting specific glycan structures (49). In this regard, it is noteworthy that elegant experiments by Román-Fernández and colleagues have shown PODXL to be a decoy receptor for galectin-3 which modifies its ability to alter integrin-dependent tumor cell invasion (12). However, it remains to be determined if this interaction also influences the immune-cold status of tumors *in vivo*. Thus, the link between altered PODXL glycosylation and poor infiltration of immune cells in tumors could be mediated by several mechanisms including a general alteration in the glycosylation profile of PODO447-expressing tumors leading to an array of new interactions between lectins and their glycan ligands or a direct interaction between a specific lectin and the PODO447 epitope. These could serve to either directly shield tumors from immune detection and/or recruit a rare subset of myeloid or stromal suppressor cells that alter the tumor environment and lead to a cold phenotype. Alternatively, the alterations in glycosylation could reflect more general changes in the metabolic activity of tumors with downstream effects on immune recognition (50). Accordingly, the characterization of the mechanisms linking PODO447 epitope expression and tumor immune phenotype and patient outcome is a top priority of future studies. Because the expression of this epitope by only a subset of tumor cells is predictive, we are focusing our attention on the production of soluble mediators selectively by PODO447^-^ and PODO447^+^ HGSOC tumors and also the possibility that PODO447 epitope endows cells with stem cell properties that are similarly at play during embryonic development and organogenesis; two scenarios known to proceed in the absence of immune-driven inflammation. Regardless of the mechanisms, we have previously shown that mice bearing PODO447^+^ xenografted tumors respond well to PODO447-ADCs and that this consistently leads to loss of expression of the PODXL core polypeptide rather than a more subtle down modulation of glycosylation of the PODO447 epitope (16). Because we and others have shown that loss of the PODXL core polypeptide expression cripples tumor growth (10–12) these data suggest that a PODO447-ADC targeted strategy could prove highly effective with a lower risk of immune escape.

While other markers, such as CA125, MSLN, FOLRA or trophoblast antigen 2 (TROP2) (30–36), are high priority candidates for the development of immune-targeting therapies to treat HGSOC, we show that PODO447 epitope has several key characteristics that distinguish it as an interesting alternative. First, PODO447 is exquisitely tumor-specific, reducing the risk of side-effect (45–47, 51). Second, the PODXL core protein has an essential role in primary and metastatic tumor cell function (10–12). Because targeting the PODO447 epitope has been shown to down regulate core protein expression this bodes well for a crippling effect on tumor growth and dissemination. Finally, despite not being express on all HGSOC, the data presented here showing a tight correlation with expression by even a subset of cells and immune-cold status suggest targeting this epitope could potentially activate an anti-cancer immune response. This therapeutic ‘trigger’ could, in turn, allow complementary treatments (like ICI) to be more effective in treating difficult-to-eliminate tumors. We are currently at the pre-clinical stage of developing multiple therapeutic strategies to target PODO447-expressing tumors, including ADC, radioisotope conjugates and cell therapy (eg, CAR T cells). It will be important to develop approaches (eg, liquid biopsies) to quickly and efficiently determine which patients express this epitope and would be likely to benefit from these therapies.

In conclusion, in this study, we demonstrated that the PODO447 epitope is minimally impacted by chemotherapy treatments and similarly expressed across different anatomical locations. We also showed a negative correlation between PODO447 expression and tumor infiltration by T cells and CD20^+^ cells. These results, in combination with our previous demonstration of the presence of this epitope only on a tumor-glycoform of PODXL, suggest that the PODO447 epitope is an excellent candidate for targeted cancer immunotherapies such as CAR T cells, T cell engagers or ADC.

## Data availability statement

The raw data supporting the conclusions of this article will be made available by the authors, without undue reservation.

## Ethics statement

The studies involving humans were approved by The University of British Columbia Research Ethics Review Board. The studies were conducted in accordance with the local legislation and institutional requirements. The participants provided their written informed consent to participate in this study.

## Author contributions

JB: Conceptualization, Formal analysis, Investigation, Methodology, Writing – original draft, Validation, Writing – review & editing. MH: Supervision, Writing – original draft, Writing – review & editing. PD: Methodology, Writing – review & editing. DH: Investigation, Methodology, Writing – review & editing. ST: Investigation, Methodology, Validation, Writing – review & editing. AB: Investigation, Writing – review & editing. JS: Investigation, Writing – review & editing. MW: Investigation, Writing – review & editing. KZ: Investigation, Writing – review & editing. KM: Investigation, Methodology, Writing – review & editing. CG: Resources, Writing – review & editing. AM: Resources, Writing – review & editing. DH: Methodology, Resources, Writing – review & editing. BN: Resources, Writing – review & editing. CR: Funding acquisition, Supervision, Writing – original draft, Writing – review & editing. KM: Funding acquisition, Resources, Supervision, Writing – original draft, Writing – review & editing.

## Supplementary material

Supplementary Figure 1 Association between the immune infiltrates and PODO447 or PODO83 expression. **(A)** Scatter plot with median comparing the number of CD4^+^, CD8^+^, CD3^+^ T cells and CD20^+^ cells in the stroma (upper panel) or in tumor (bottom panel) areas in HGSOC tumors that express low (tumor H-score <5), medium (tumor H-score 5 to 50) or high level (tumor H-score >50) of the PODO447 epitope. **(B)** Spearman’s correlation between PODO83 tumor H-score and the number of CD4, CD8 and CD3 T cells and CD20^+^ cells in the stroma area (upper panel) or in the tumor area (bottom panel). Data are from Cohort D. *= p<0.05 using Kruskal-Wallis test; Only p-values below 0.1 are displayed.

Supplementary Figure 2 Association of PODO447 epitope expression and immune phenotype in Cohort D. **(A)** Segregation strategy used to categorize tumors as immune-desert, immune-excluded or immune-inflamed based on the number of CD20^+^ and CD8^+^ cells localized in the tumor and the stroma areas. **(B)** Bar plot with median comparing PODO447 or PODO83 tumor H-score between the three immune-phenotype groups. Kruskal-Wallis test with Dunn’s multiple comparisons test was performed leading to no significant differences. **(C)** Pie-chart showing the proportions of immune-desert, immune-excluded or immune-inflamed tumors within the PODO447^low/med^ group (tumor H-score <50) or within the PODO447^high^ group (tumor H-score >50).

Supplementary Figure 3 Association between the immune infiltrate and CA125, MSLN or FOLRA. Spearman’s correlation between the percentage of tumor positive cells for **(A)** CA125, **(B)** MSLN or **(C)** FOLRA and the number of CD4^+^, CD8^+^, CD3^+^ T cells and CD20^+^ cells in the stroma area (upper panel) or the tumor area (bottom panel). Data are from Cohort D.

Supplementary Figure 4 Association of PODO447 epitope expression and immune phenotype in Cohort A. **(A)** Segregation strategy used to categorize tumors as immune-desert, immune-excluded or immune-inflamed based on the number of CD20^+^ and CD8^+^ cells localized in the tumor and the stroma areas. **(B)** Bar plot with median comparing PODO447 or PODO83 tumor H-score between the three immune-phenotype groups. Kruskal-Wallis test with Dunn’s multiple comparisons test was performed leading to no significant differences. **(C)** Pie-chart showing the proportions of immune-desert, immune-excluded or immune-inflamed tumors within the PODO447^low/med^ group (tumor H-score <50) or within the PODO447^high^ group (tumor H-score >50).
