# Supplementary figures and images for: A tumor-restricted glycoform of podocalyxin is a highly selective marker of immunologically cold high-grade serous ovarian carcinoma
Source: Front Oncol. 2023 Dec 21;13:1286754. doi: 10.3389/fonc.2023.1286754 (PMC10771318; doi:10.3389/fonc.2023.1286754)

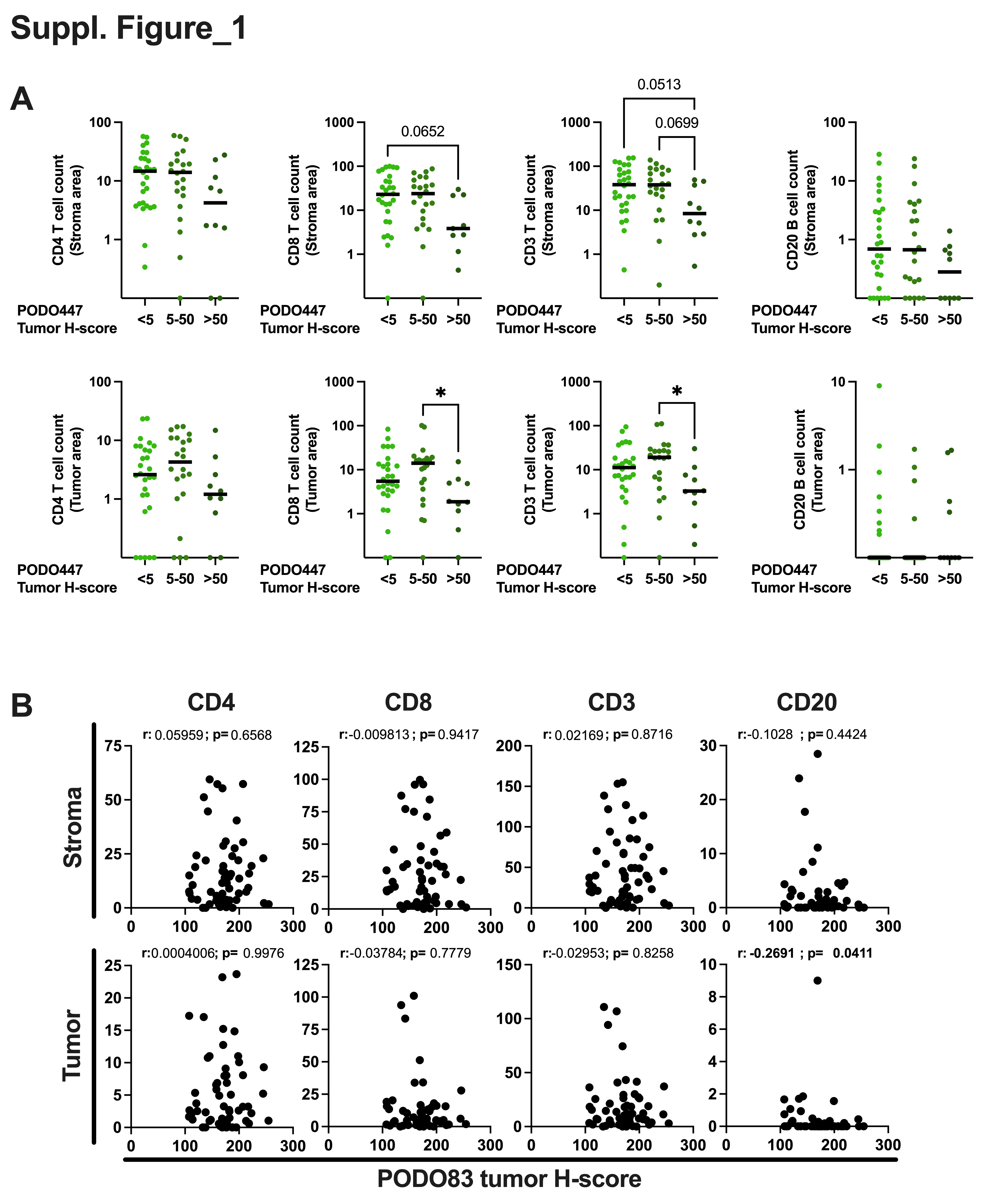

Supplement: Supplementary Figure 1 — Association between the immune infiltrates and PODO447 or PODO83 expression. (A) Scatter plot with median comparing the number of CD4+, CD8+, CD3+ T cells and CD20+ cells in the stroma (upper panel) or in tumor (bottom panel) areas in HGSOC tumors that express low (tumor H-score <5), medium (tumor H-score 5 to 50) or high level (tumor H-score >50) of the PODO447 epitope. (B) Spearman’s correlation between PODO83 tumor H-score and the number of CD4, CD8 and CD3 T cells and CD20+ cells in the stroma area (upper panel) or in the tumor area (bottom panel). Data are from Cohort D. *= p<0.05 using Kruskal-Wallis test; Only p-values below 0.1 are displayed. [file Image_1.tiff]

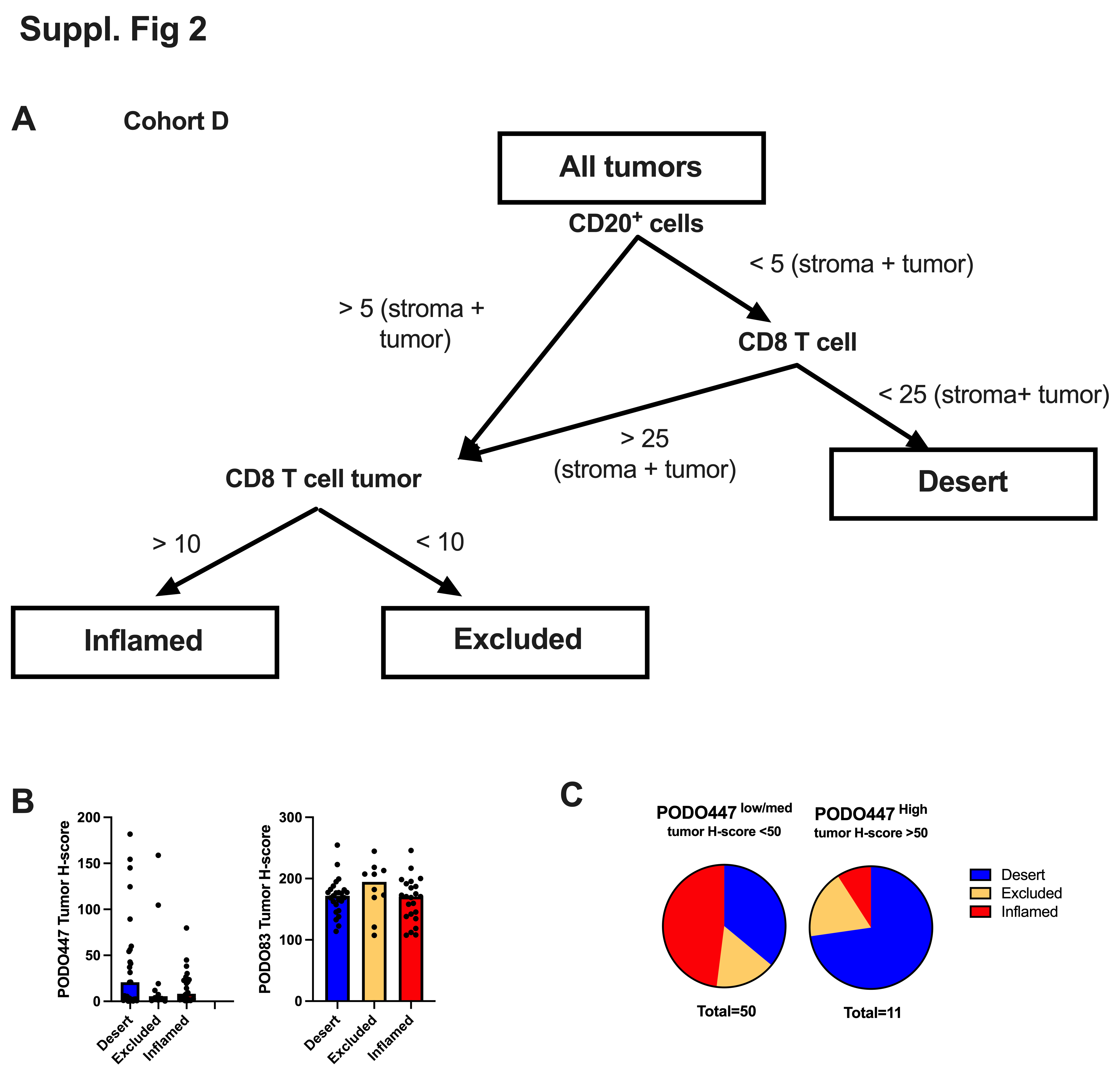

Supplement: Supplementary Figure 2 — Association of PODO447 epitope expression and immune phenotype in Cohort D. (A) Segregation strategy used to categorize tumors as immune-desert, immune-excluded or immune-inflamed based on the number of CD20+ and CD8+ cells localized in the tumor and the stroma areas. (B) Bar plot with median comparing PODO447 or PODO83 tumor H-score between the three immune-phenotype groups. Kruskal-Wallis test with Dunn’s multiple comparisons test was performed leading to no significant differences. (C) Pie-chart showing the proportions of immune-desert, immune-excluded or immune-inflamed tumors within the PODO447low/med group (tumor H-score <50) or within the PODO447high group (tumor H-score >50). [file Image_2.tiff]

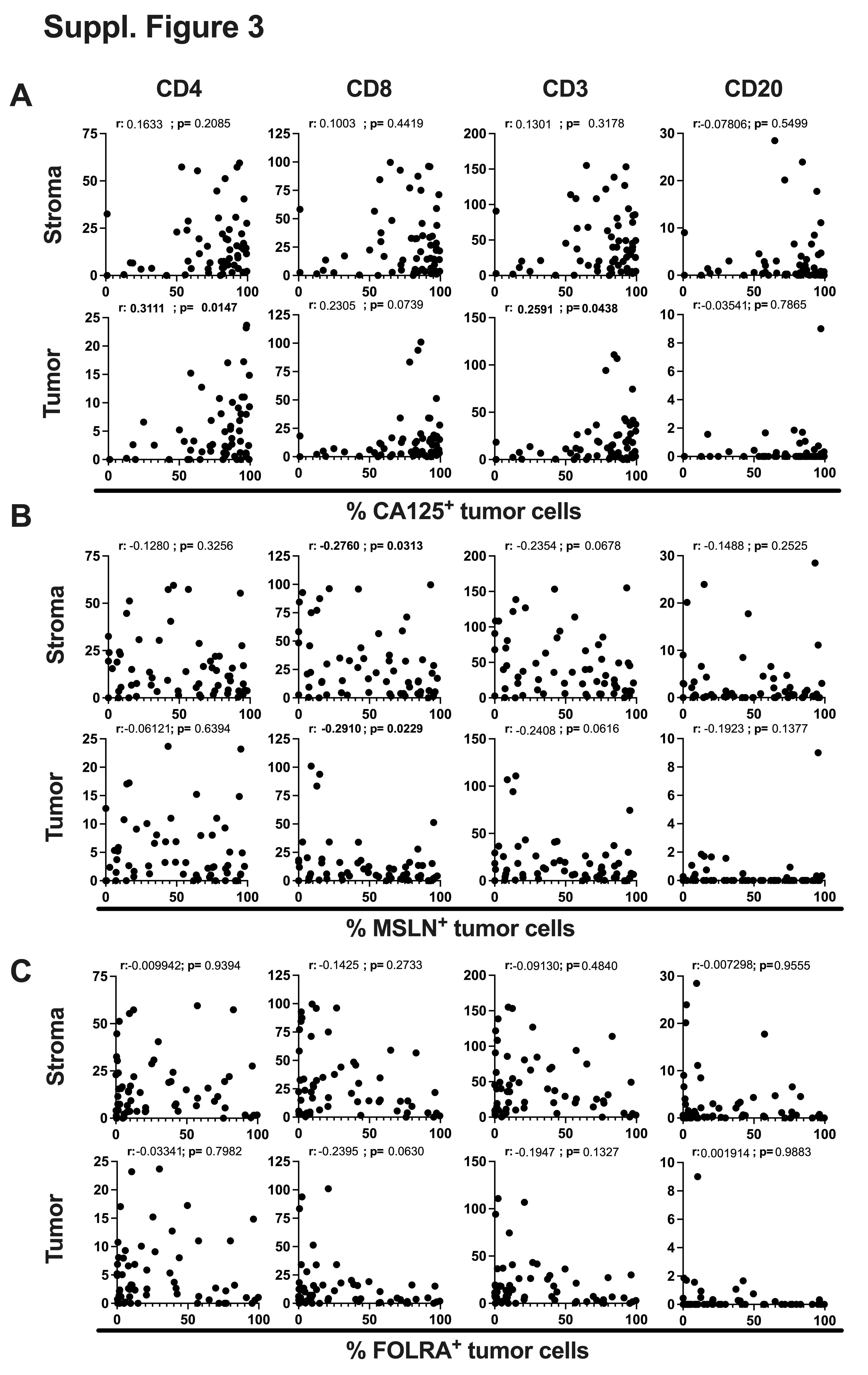

Supplement: Supplementary Figure 3 — Association between the immune infiltrate and CA125, MSLN or FOLRA. Spearman’s correlation between the percentage of tumor positive cells for (A) CA125, (B) MSLN or (C) FOLRA and the number of CD4+, CD8+, CD3+ T cells and CD20+ cells in the stroma area (upper panel) or the tumor area (bottom panel). Data are from Cohort D. [file Image_3.tiff]

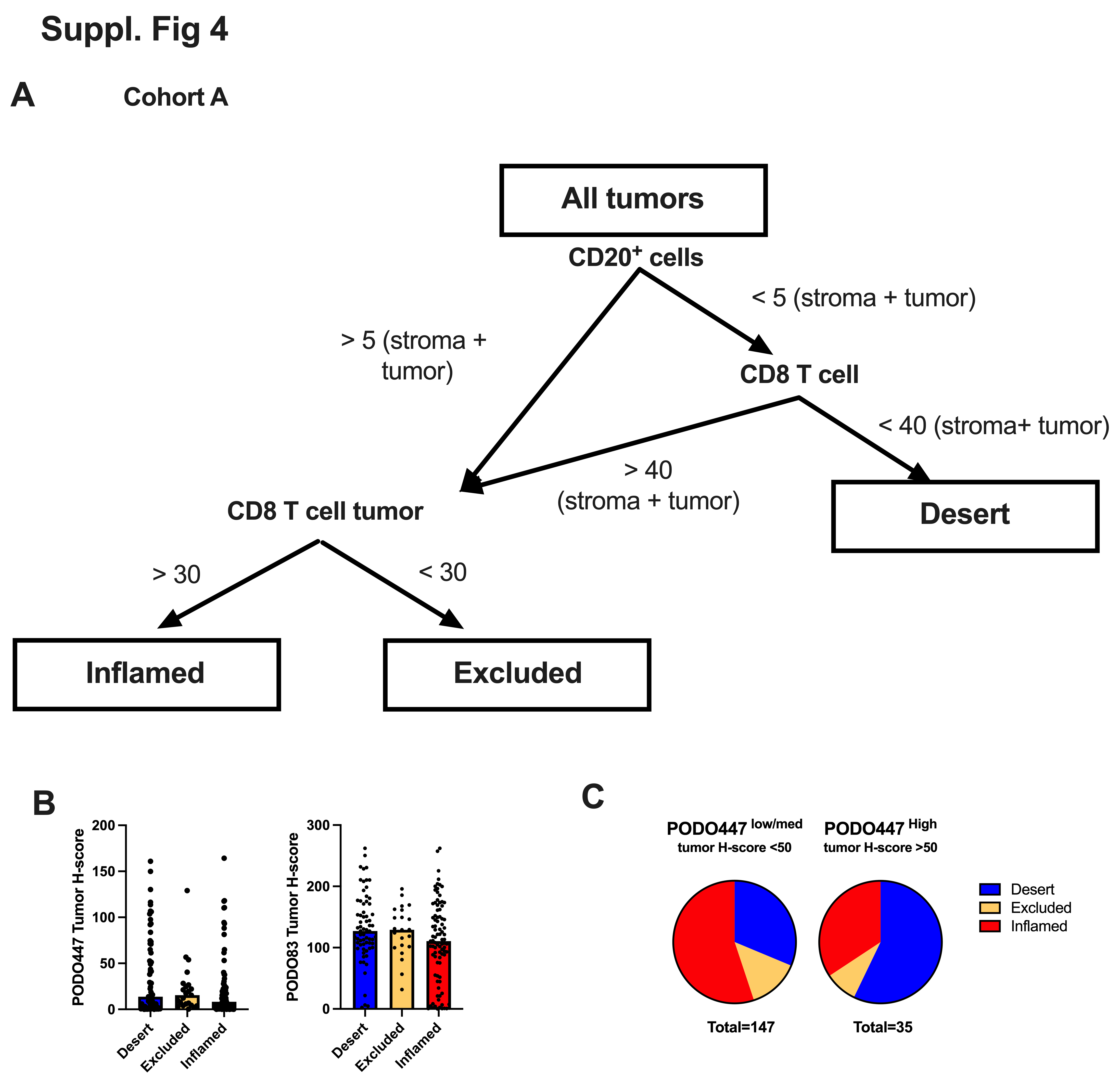

Supplement: Supplementary Figure 4 — Association of PODO447 epitope expression and immune phenotype in Cohort A. (A) Segregation strategy used to categorize tumors as immune-desert, immune-excluded or immune-inflamed based on the number of CD20+ and CD8+ cells localized in the tumor and the stroma areas. (B) Bar plot with median comparing PODO447 or PODO83 tumor H-score between the three immune-phenotype groups. Kruskal-Wallis test with Dunn’s multiple comparisons test was performed leading to no significant differences. (C) Pie-chart showing the proportions of immune-desert, immune-excluded or immune-inflamed tumors within the PODO447low/med group (tumor H-score <50) or within the PODO447high group (tumor H-score >50). [file Image_4.tiff]
