# Supplementary material for: A tumor-restricted glycoform of podocalyxin is a highly selective marker of immunologically cold high-grade serous ovarian carcinoma
Source: Front Oncol. 2023 Dec 21;13:1286754. doi: 10.3389/fonc.2023.1286754 (PMC10771318; doi:10.3389/fonc.2023.1286754)
Supplement: Supplementary file 5 [file Table_1.docx]

**Supplementary Table S1: PODO83 and PODO447 staining methods – additional information.**

| Cohort | | GPEC 08-001 | CHUM -THT3 | CHUM  (pre/post-chemo) | IROCPROS5 |
| --- | --- | --- | --- | --- | --- |
| Identification | | A | B | C | D |
| PODO83 | primary mAb | Rb αPODO83  2 µg/mL; O/N 4°C | Rb αPODO83-BIO  2 μg/ml; O/N 4°C | Rb αPODO83-BIO  2 μg/ml; O/N 4°C | Rb αPODO83  1 µg/mL; 30 min RT |
|  | secondary mAb | α Rb-BIO*  2 μg/ml; 30 min RT | Ø | Ø | MACH 2 Rb HRP-polymer (30 min RT) |
| PODO447 | primary mAb | Rb αPODO447  5 µg/mL; O/N 4°C | Rb αPODO447-BIO  1 μg/ml; O/N 4°C | Rb αPODO447-BIO  1 μg/ml; O/N 4°C | Rb αPODO447  2.5 µg/mL; 30 min RT |
|  | secondary mAb | αRb-BIO*  2 μg/ml; 30 min RT | Ø | Ø | MACH 2 Rb HRP-polymer (30 min RT) |

Ø – not required, mAb = monoclonal antibody; O/N = overnight; RT = room temperature. Rb = rabbit, BIO = biotin conjugated

*Southern Biotech, #6440-06

**Supplementary Table S2: Antibody information for multiplex staining**

| Multiplex Panels | Primary antibodies | | | Secondary antibodies  (From Biocare) | Detection reagents |
| --- | --- | --- | --- | --- | --- |
|  | Antigen | Clone | Conc. |  |  |
| Cohort A: Fluorescent Opal multiplex | PODO447 | n/a | 1:500 | Streptavidin-HRP | Opal 520 |
|  | PODO83 | n/a | 1:25,000 | Mach2-Rb-HRP | Opal 650 |
|  | CD20 | L26 | 1:200 | Mach2-Ms-HRP | Opal 570 |
|  | CD8 | C8/144B | 1:300 | Mach2-Ms-HRP | Opal 620 |
|  | Pan-CK | AE1/AE3+8/18 | 1:450 | Mach2-Ms-HRP | DIG |
|  | Opal 780 Anti-DIG | n/a | 1:100 | n/a | n/a |
| Cohort D: IHC | CD3 | M3074 | 1:500 | Mach 2 Double Stain 2 | intelliPATH^TM^ Ferengi Blue |
|  | CD8 | 108M-94 | 1:250 |  | intelliPATH^TM^ DAB |
|  | CD20 | CM004 | 1:300 | Mach 2 Ms-AP Polymer | intelliPATH^TM^ Warp Red |
| Cohort D: Fluorescent Opal multiplex | Pan-CK | CM162A | 1:100 | Mach 2 Ms-HRP | Opal 690 |
|  | CA125 | CM101A | 1:150 | Mach 2 Ms-HRP | Opal 570 |
|  | MSLN | MA5-11918 | 1:20 | Mach 2 Ms-HRP | Opal 620 |
|  | FOLRA | FRALPHA-L-CE | 1:150 | Mach 2 Ms-HRP | Opal 650 |

n/a: non-applicable; Conc. : concentration; Ms : mouse; Rb : Rabbit
