# Supplementary material for: A tumor-restricted glycoform of podocalyxin is a highly selective marker of immunologically cold high-grade serous ovarian carcinoma
Source: Front Oncol. 2023 Dec 21;13:1286754. doi: 10.3389/fonc.2023.1286754 (PMC10771318; doi:10.3389/fonc.2023.1286754)
Supplement: Supplementary file 6 [file DataSheet_1.pdf]

1 Supplementary Methods

1.1 Brightfield IHC

1.1.1 Cohort A: (Modified from Canals Hernaez et al. J Immunother Cancer. 2020 (Canals Hernaez et al., 2020))

Slides were deparaffinized and rehydrated using xylenes and ethanol gradient and rinsed in distilled water. Antigen retrieval was performed by heating slides in citrate buffer (pH=6) at 90°C for 30 min. Slides were washed in PBS, incubated for 30 min in blocking solution (PBS, 5% donkey serum, 1% BSA, 0.2% Triton X-100, 0.05% Tween 20), and incubated in primary mAb solution overnight at 4°C. Slides were then washed with TBST, incubated with secondary Ab in blocking solution, and washed again with TBST. Endogenous peroxidase was blocked with 0.3% hydrogen peroxide in methanol for 30 min at room temperature (RT) prior to rinsing with PBS. Signal was amplified using Vectastain® Elite® ABC HRP Kit (Vector Laboratories, #PK-6100) according to manufacturer's instructions, and then washed with PBS and visualized using a DAB peroxidase (HRP) Substrate Kit (Vector Laboratories, #SK-4100) following the manufacturer's recommendations. Each slide was incubated with DAB solution for 1-5 min until strong signal appeared, then the reaction was stopped by dilution with PBS. Slides were washed with PBS, distilled water, stained in hematoxylin (VWR, #10143- 150) for 12 s and rinsed with tap water. Staining was fixed in Bluing Solution (0.1% sodium bicarbonate) for 1 min and rinsed in distilled water prior to dehydration in graded alcohols and xylenes. Slides were mounted using Permount solution (Fisher Scientific, #SP15100).

1.1.2 Cohort B & C:

Staining protocols performed on Cohort B and C TMA slides were the same as Cohort A except that the antigen retrieval step for the PODO447 staining was performed in a pH 9 buffer (H_2O, 10mM Tris-Base, 1mM EDTA and 0.05% Tween-20), washing steps before the incubation with the PODXL mAbs were done in PBS and in PBS-T (PBS, 0.05% Tween-20) after the incubation, and finally as the PODXL mAbs were already biotinylated, no secondary Ab step was needed.

1.1.3 Cohort D: (Modified from Smazynski et al. Gynecol Oncol. 2020 (Smazynski et al., 2020) and Banville et al. Gynecol Oncol. 2021 (Banville et al., 2021))

Tissue sections were incubated overnight at 37°C, deparaffinized and rehydrated using xylenes and an ethanol gradient and rinsed in distilled water. Antigen retrieval was performed in Diva decloaker reagent (Biocare, Pacheco, CA) in a Biocare decloaking chamber (110°C for 15 min), and slides were loaded into an Intellipath FLX Autostainer. Unless otherwise mentioned, staining was performed at RT with Biocare reagents (Biocare Medical, Pacheco, CA). Slides were treated with Peroxidase-1 for 5 min and Background Sniper for 10 min. For PODO83 and PODO447 IHC staining, primary mAbs were diluted in DaVinci Green and applied for 30 min followed by 30 min incubation with Mach2 Rabbit-HRP polymer. Each slide was incubated with DAB solution for 5 min. For the immune panel staining, slides were manually stained with a cocktail of primary mAbs against CD3 and CD8 with DaVinci Green diluent for 30 min. Slides were then re-loaded onto the Intellipath and stained with Mach2 Double Stain 2 for 30 min, followed by intelliPATH™ Ferengi Blue chromogen for 8 min, and intelliPATH™ DAB chromogen for 5 min. To strip remaining antibody, slides were manually rinsed with distilled water and incubated with SDS-glycine (pre-warmed to 50°C, pH 2.0) for 45 min with agitation. Slides were then washed with TBS automation

wash buffer then manually stained with CD20 antibody diluted in DaVinci Green diluent for 30 min. Slides were then re-loaded onto the Intellipath and sequentially incubated with Mach2 Mouse-AP Polymer for 30 min, intelliPATH™ Warp Red chromogen for 7 min. CAT hematoxylin diluted 1:5 with distilled water was added for 5 min at RT. Stained slides were washed with water, air-dried and cover-slipped using Ecomount.

1.2 Fluorescent Opal Multiplex Staining

1.2.1 Cohort D: 4-colour immunofluorescent panel (modified from Banville et al. Gynecol Oncol. 2021 (Banville et al., 2021))

All reagents used were from Biocare with the exception of Opal dyes (from Akoya Biosciences) and all steps were performed at RT unless otherwise mentioned. TMA slides were baked at 37°C overnight, deparaffinized manually, and then post-fixed in 10% neutral buffered formalin (Sigma Aldrich) for 20 min. Slides were then thoroughly rinsed in distilled water and subjected to antigen retrieval in a decloaking chamber with Diva Decloaker reagent for 15 min at 110°C followed by 10 min of cooling. Slides were then washed with distilled water and loaded onto the Intellipath FLX Autostainer to perform endogenous peroxidase and non-specific blocking using Peroxidased-1 (5 min) and Background Sniper (10 min). The anti-FOLRA antibody was diluted in DaVinci Green Diluent and manually added to slides for a 30-min incubation, followed by Mach 2 Mouse-HRP Polymer and then Opal 650 for 10 min each (on Intellipath). Slides were then removed from the Intellipath, rinsed with distilled water, and subjected to antigen retrieval with AR6 (Akoya Biosciences) using a microwave (1 min at max power followed by 15 min at power level 1). Slides were then cooled for 15 min followed by washing with distilled water and then re-loaded back onto the Intellipath for a second round of endogenous and non-specific blocking with

Peroxidased-1 and Background sniper (5 min each). The anti-CA125 antibody diluted in Renoir Red was then manually added to slides for a 30-min incubation, followed by Mach 2 Mouse-HRP Polymer and then Opal 570 for 10 min each. The previously described antigen retrieval steps were then repeated with AR6 followed by a third round of endogenous and non-specific blocking as described above. Anti-MSLN antibody diluted in DaVinci Green was then manually added to slides for a 30-min incubation, followed by Mach 2 Mouse-HRP Polymer and then Opal 620 for 10 min each. A final round of antigen retrieval and blocking was performed as previously described. Anti-pan-CK antibody diluted in DaVinci Green was then manually added to slides for a 30-min incubation, followed by Mach 2 Mouse-HRP Polymer and then Opal 690 for 10 min each. DAPI (diluted in automation wash buffer with Tween) was then added to slides for 5 min. Slides were then rinsed with distilled water, air dried, and cover-slipped with Fluoro Care Anti-Fade Mountant.

1.2.2 Cohort A: 5-color panel

All reagents used were from Leica Biosystems (apart from Opal dyes, antibody diluents and protein block; Akoya Biosciences, Peroxidase-1 and HRP polymers; Biocare). The staining was performed using the Fully automated BOND RX Research Stainer by Leica Biosystems. The deparaffinization/rehydration step was performed using the Leica Dewax reagent followed by a gradient of ethanol to TBS based buffer (Buffer 1 from Leica) and then the slides were post-fixed in 10% Neutral Buffered Formalin. Antigen retrieval was performed using the BOND Epitope Retrieval Solution 2 for 40 min at 95°C. Endogenous peroxidase blocking was performed using Peroxidased-1 reagent for 6 min and endogenous protein blocking was performed by incubating for 10 min with the opal antibody diluent, then 30 min with Avidin and 30 min with Biotin

solutions. This was followed by successive rounds of incubation with the primary antibody (30 min, 37°C), HRP secondary antibody (10 min, 37°C) and Opal Fluorophore (10 min, 37°C) followed by a denaturation step using the BOND Epitope Retrieval Solution 1 for 20 min at 95°C and a subsequent step of endogenous protein and endogenous peroxidase blocking (performed as mentioned earlier). The staining sequence was PODO447 first followed by PODO83, CD20, CD8 and finally pan-CK and the reagents used as well as the concentration are described in suppl. Table S2. Following all the staining cycles, nuclei counterstain was performed using DAPI. Slides were then rinsed with distilled water, air dried, and cover-slipped with ProLong™ Diamond Antifade Mountant (ThermoFisher). Mounted slides were cured for 24 h at RT in the dark prior to scanning. Long term storage of mounted sides at -20°C.

2 References

Bankhead, P., Loughrey, M.B., Fernández, J.A., Dombrowski, Y., Mcart, D.G., Dunne, P.D., Mcquaid, S., Gray, R.T., Murray, L.J., Coleman, H.G., James, J.A., Salto-Tellez, M., and Hamilton, P.W. (2017). QuPath: Open source software for digital pathology image analysis. *Sci Rep* 7**,** 16878.

Banville, A.C., Wouters, M.C.A., Oberg, A.L., Goergen, K.M., Maurer, M.J., Milne, K., Ashkani, J., Field, E., Ghesquiere, C., Jones, S.J.M., Block, M.S., and Nelson, B.H. (2021). Co-expression patterns of chimeric antigen receptor (CAR)-T cell target antigens in primary and recurrent ovarian cancer. *Gynecol Oncol* 160**,** 520-529.

Canals Hernaez, D., Hughes, M.R., Dean, P., Bergqvist, P., Samudio, I., Blixt, O., Wiedemeyer, K., Li, Y., Bond, C., Cruz, E., Köbel, M., Gilks, B., Roskelley, C.D., and Mcnagny, K.M. (2020). PODO447: a novel antibody to a tumor-restricted epitope on the cancer antigen podocalyxin. *J Immunother Cancer* 8.

Smazynski, J., Hamilton, P.T., Thornton, S., Milne, K., Wouters, M.C.A., Webb, J.R., and Nelson, B.H. (2020). The immune suppressive factors CD155 and PD-L1 show contrasting expression patterns and immune correlates in ovarian and other cancers. *Gynecol Oncol* 158**,** 167-177.
